# A novel family of expression vectors with multiple affinity tags for wheat germ cell-free protein expression

**DOI:** 10.1186/s12896-020-00610-5

**Published:** 2020-03-14

**Authors:** Szilvia Krisztina Nagy, Brigitta Margit Kállai, Judit András, Tamás Mészáros

**Affiliations:** Department of Medical Chemistry, Molecular Biology and Pathobiochemistry, Semmelweis University, 37-47 Tűzoltó Street, Budapest, H-1094 Hungary

**Keywords:** Cell-free, Protein expression, Wheat germ, TEV, Affinity tag, FLAG, Halo

## Abstract

**Background:**

Cell-free protein expression has become a widely used alternative of in vivo, cell-based systems in functional and structural studies of proteins. The wheat germ-based method outstands from the commercially available eukaryotic in vitro translation systems by its flexibility, high translation efficiency and success rate of properly folded eukaryotic protein synthesis. The original T7 promoter containing pEU3-NII vector was improved previously by addition of a ligation-independent cloning site, His_6_- and GST-tags, and a TEV protease cleavage site to facilitate the creation of recombinant plasmids, permit affinity purification, and enable production of purified, tag-free target proteins, respectively.

**Results:**

Here, we describe a further development of pEU3-NII vector by inserting the rare-cutting, NotI restriction enzyme cleavage site to simplify vector linearization step prior to in vitro transcription. Additionally, His_12_, FLAG, and Halo affinity tag coding vectors have been created to increase detection sensitivity, specificity of interaction studies, and provide covalently linkable ligands for pull-down assays, respectively. Finally, the presented GST-His_6_, and GST-biotin double-tagging vectors could broaden the range of possibilities of protein-protein interaction studies.

**Conclusions:**

The new generation of pEU3-NII vector family allows a more rapid production of translationally active mRNA and wheat germ cell-free expression of target proteins with a wide variety of affinity tags thus enables designing flexible and diverse experimental arrangement for in vitro studies of proteins.

## Background

Although straightforward and cost-effective production of properly folded proteins is a general starting point of protein studies, the accomplishment of this step could be a challenging task, especially in the case of eukaryotic proteins. Various cell-free in vitro translation systems have been developed to subdue this obstacle and amongst them, the wheat germ-based approach systems seem to be the choice of method for high-throughput eukaryotic protein production [1, 2].

The eukaryotic translation machinery requires an extensively modified mRNA, namely 5′ methylguanosine capping and 3′ poly(A) tailing. In order to evade these complex posttranscriptional modifications, a wheat germ in vitro translation compatible vector has been constructed with the cap replacing, translational enhancer Ω sequence from the tobacco mosaic virus and an additional GAA triplet at the 5′-end (GAA Ω) [3]. The same group also demonstrated that translation did not strictly depend on the presence of poly(A) tail, it could be replaced by a 1626 nucleotide long sequence, thus any 3′-UTR sequence of minimum length could substitute the poly(A) tail. To produce a linear DNA template for T7 RNA polymerase [4], the vector construct is cut by a restriction endonuclease at a specific site, forming a 3′-UTR of the appropriate length. The above-mentioned observations resulted in the creation of pEU3-NII and pEU-E01 vectors harbouring T7 and SP6 promoters, respectively, that could be efficiently applied for in vitro production of translationally active mRNAs.

However, their application was hindered by restriction endonuclease-based insertion of the coding sequence of interest and lack of protein purification aiding affinity tags. To increase their practicality, we have redesigned the original vectors and replaced the multicloning site by a ligation-independent cloning (LIC) compatible sequence and inserted tobacco etch virus (TEV) protease cleavable hexahistidine (His_6_), and glutathione S-transferase (GST) coding sequences to aid the construction of recombinant plasmids and the purification of in vitro translated proteins [5]. The improved vectors (pEU3-NII-HLIC and pEU3-NII-GLIC) could shorten the timeline of ‘from DNA to purified functional protein’ to a week and we successfully applied them for synthesizing dozens of eukaryotic proteins of various organisms [6–12].

The emergence of recombinant affinity tags was a significant improvement of the heterologous protein producing systems since they streamlined purification of the protein of interest and augmented the solubility of expressed proteins in many cases. Since the first application of affinity tags several protein labelling techniques have been invented mainly to meet the demands of downstream applications of synthesized proteins [13].

In order to extend the versatility of wheat germ in vitro translation systems, we set out to expand the pEU3-NII vector family by providing various protein affinity tags, which could aid pull-down assays, interaction analysis, and enzymatic functional studies of synthesized target proteins. Firstly, a rare-cutting restriction endonuclease (NotI) cleavage site [14] was inserted into the pEU3-NII vectors to allow the straightforward plasmid linearization prior to in vitro mRNA transcription. We demonstrate that protein detection sensitivity is elevated by using our double-His_6_ label coding vector [15]; while the FLAG-tag offering plasmid ensures a highly selective detection of labelled protein [16, 17]. Additionally, we show that our HaloTag [18] encoding vector is optimal for producing proteins that could be covalently linked to affinity beads of pull-down assays. Finally, we provide the vectors with GST-His_6_ and GST-biotin affinity tags with TEV cleavable GST to ease the purification of labelled recombinant proteins.

## Results and discussion

The pEU3-NII-HLIC and pEU3-NII-GLIC vectors were extensively used for synthesizing a great variety of recombinant proteins by the wheat germ in vitro translation, therefore a next improvement was implemented to make the procedure even more straightforward and adaptable.

The vector constructs are linearized by restriction endonuclease in advance of in vitro mRNA synthesis by T7 RNA polymerase. Cleavage sites present in the ORF of the genes of interest are not suitable, thus a generally usable, rare-cutting restriction endonuclease cleavage site, NotI (GCGGCCGC) was inserted by in vitro mutagenesis in both vectors 2424 bp downstream from the LIC site (pEU3-NII-HLICNot and pEU3-NII-GLICNot). That resulted in a more straightforward sample handling in case of multiple plasmid constructs and mRNAs with identical, appropriate size 3′-UTR were produced.

**Fig. 1:**
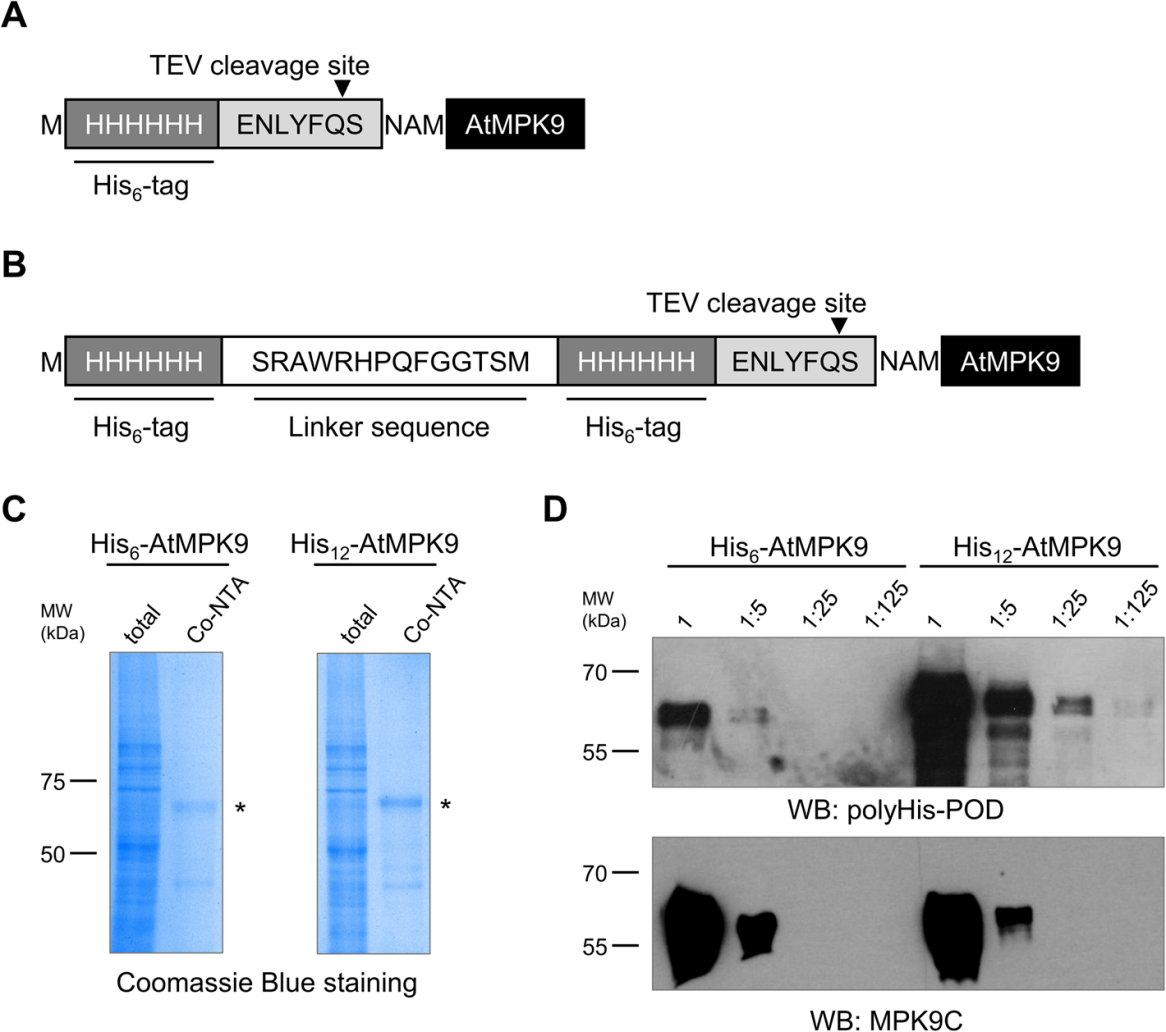
IMAC purification and Western blot analysis of His_6_-AtMPK9 and His_12_-AtMPK9 proteins. **a**, **b** Amino acid sequence of the tagging regions of His_6_- and His_12_-labeled AtMPK9 vector constructs. **c** The in vitro translated His_6_-AtMPK9 and His_12_-AtMPK9 were purified with Co-NTA affinity beads, and the total translation mixtures and purified fractions were separated with SDS-PAGE and visualized by Coomassie Blue staining. The purified AtMPK9 proteins are indicated by asterisks. **d** Serial five-fold dilutions of 1 μl of total translation mixtures were studied by Western blotting using anti-polyHis-POD and anti-MPK9C. The molecular weight markers are shown in kDa

Probably, the most popular label of recombinant proteins is the amino acid stretch of six histidines (His_6_); however, its usability is hindered by the relatively low binding affinity of coordinating histidine residues to metal ions and promiscuity of His_6_ selective antibodies. To mitigate these drawbacks, a double-His_6_ holding *E. coli* expression vector was constructed by Khan et al. [15]. The authors demonstrated that application of double-His_6_ improved the binding affinity of proteins to nickel-nitrilotriacetic acid (Ni-NTA) modified surfaces and increased the detectability of overexpressed proteins. Considering that characterization of weak protein-protein interactions requires sensitively detectable proteins, we constructed a vector for in vitro translation of N-terminally double-His_6_ (His_12_) labelled recombinant proteins. In order to assess the advantage of the novel plasmid construct, the coding sequence of an *Arabidopsis thaliana* mitogen-activated protein kinase (AtMPK9) was inserted into the created pEU3-NII-HxHLICNot vector by LIC method. His_6_-AtMPK9 (Fig. 1a) and His_12_-AtMPK9 (Fig. 1b) were produced by wheat germ in vitro translation, purified by immobilized metal affinity chromatography (IMAC) with Co-NTA beads and analysed by Coomassie Blue staining. The results of affinity purification demonstrated that both recombinant proteins were effectively synthesized and purified by Co-NTA beads (Fig. 1c). To test the sensitivity of protein detection, we prepared five-fold dilutions of total translation mixtures containing His_6_-AtMPK9 and His_12_-AtMPK9 proteins and analysed them by Western blot. The results showed that doubling the number of histidine residues significantly elevated the sensitivity of Western blot analysis; at least one order of magnitude smaller amount of His_12_-AtMPK9 was sufficient for the detection with anti-polyHistidine antibody in comparison to the traditionally His_6_-tagged AtMPK9 (Fig. 1d). To rule out unequal loading of the differently tagged AtMPK9 proteins, immunodetection with anti-MPK9C antibody was also performed. (Fig. 1d).

**Fig. 2:**
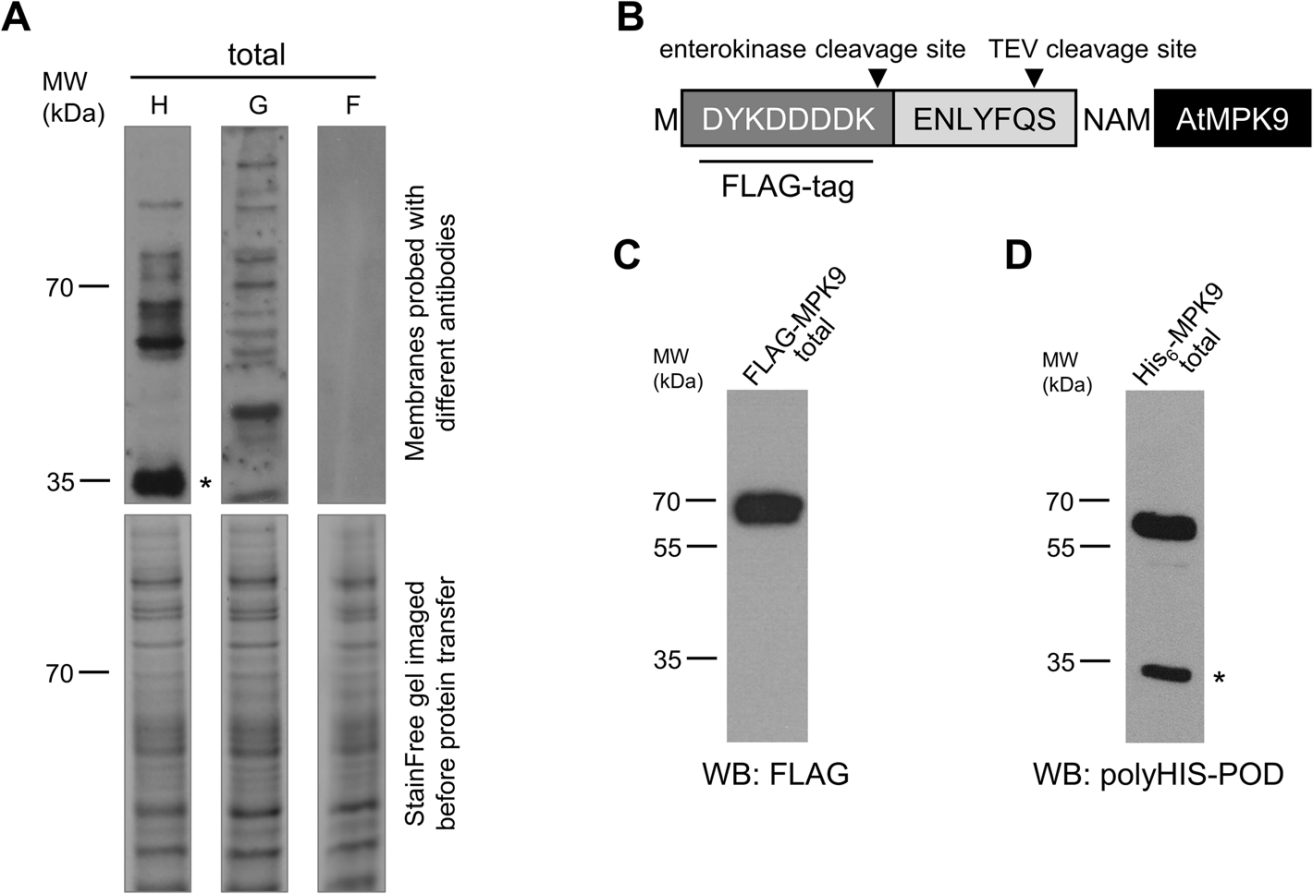
Western blot analysis of crude wheat germ protein extract and FLAG-tagged AtMPK9. **a** Total translation mixture of wheat germ extract (W7240) was separated by TGX Stain-Free gel and imaged by UV light exposure (bottom panel). Following the transfer of proteins to PVDF membrane samples were analysed by Western blotting using anti-polyHis-POD (lane H), anti-GST (lane G), and anti-FLAG (lane F) antibodies. **b** Amino acid sequence of the tagging region of FLAG-AtMPK9. **c** Total translation mixture containing FLAG-AtMPK9 was separated by SDS-PAGE and analysed by Western blotting using anti-FLAG antibody. **d** Total translation mixture containing His_6_-AtMPK9 was separated by SDS-PAGE and analysed by Western blotting using polyHis-POD antibody. The most abundant non-specific protein signal detected with anti-polyHis-POD is marked by an asterisk. The molecular weight markers are shown in kDa

Due to its small size, hydrophilic nature, and tyrosine content, the FLAG epitope tag commonly resides on the surface of the fusion protein and provides an excellent antibody binding site [17]. These properties of FLAG-tag resulted in the generation of greatly discriminating commercial antibodies and wide-spread application of this labelling system. FLAG-tagging is a rational choice of protein labelling when highly selective detection rather than purification of the synthesized protein is the priority. To assess the performance of the FLAG system in comparison to other antibodies in the wheat germ protein extract, total translation mixtures were separated by SDS-PAGE, blotted and analysed by using anti-polyHistidine, anti-GST, and anti-FLAG antibodies. Detection of protein-bound antibodies by enhanced chemiluminescence (ECL) demonstrated that anti-polyHistidine and anti-GST IgG could bind to several endogenous wheat germ proteins, while anti-FLAG antibody remarkably did not produce any signal (Fig. 2a). The undetectable background signal of FLAG antibodies with wheat germ protein extract encouraged us to develop a pEU3-NII vector with N-terminal FLAG-tag. In order to examine the superiority of the novel vector, AtMPK9 was synthesized with His_6_- and FLAG-tag (Fig. 2b) and the total translation mixtures were studied by Western blot analysis. In accordance with the previous results, the FLAG selective antibody decorated a single band of FLAG-AtMPK9 at the expected molecular weight (Fig. 2c). Although the polyHistidine antibody effectively detected His_6_-AtMPK9, an additional, endogenous protein of wheat germ extract was also clearly recognized by this antibody (Fig. 2d).

**Fig. 3:**
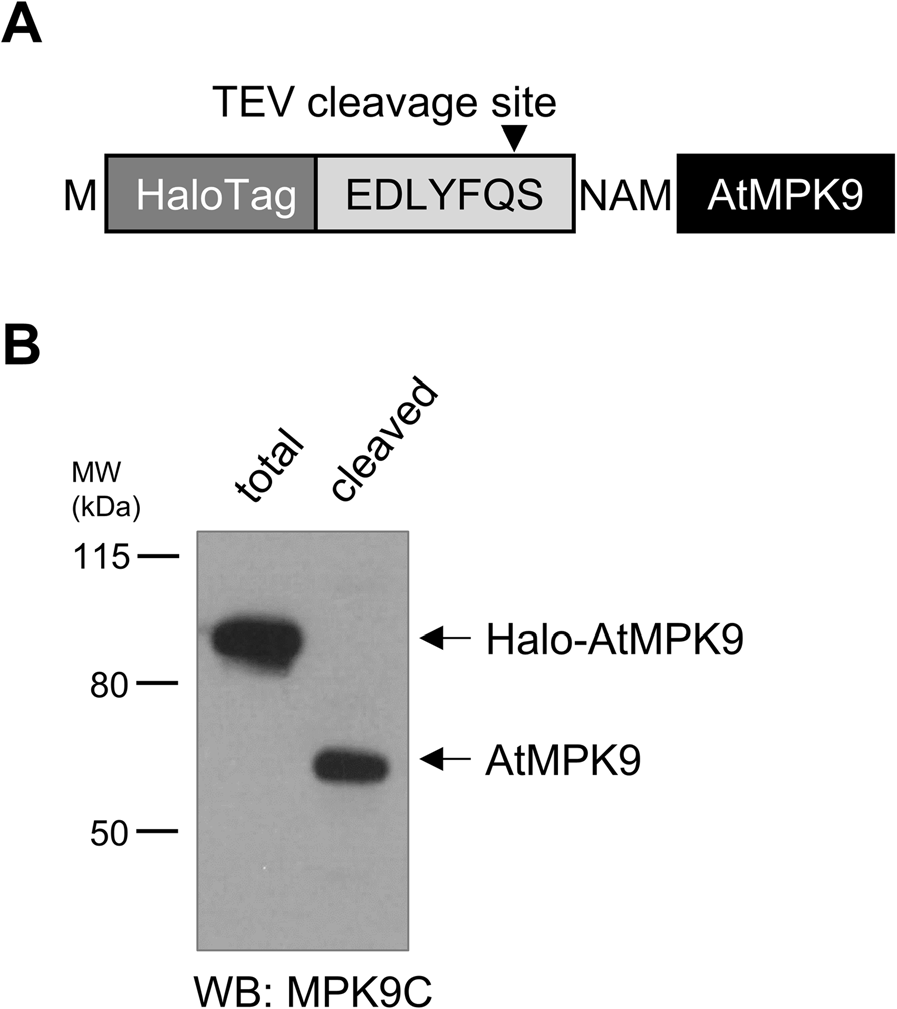
Western blot analysis of translated Halo-AtMPK9 protein. **a** Amino acid sequence of tagging region of HaloTag-labelled AtMPK9 vector construct. **b** Halo-AtMPK9 was purified from total translation mixture with HaloTag affinity beads and AtMPK9 was released from the beads by TEV protease cleavage. The samples were separated by SDS-PAGE and AtMPK9 was detected by immunoblotting with anti-MPK9C antibody. The molecular weight markers are shown in kDa

Elucidating protein function involves the understanding of dynamic cellular protein networks and the pull-down technique is a valuable tool for revealing the underlying protein-protein interactions. It is equally suitable for identification of novel interacting partners and confirmation of putative interactions. The validity of pull-down assay provided data is greatly determined by the appropriately chosen binding and washing conditions; thus, the strong ligand-bead interaction is an inevitable component of successful assays. To address the above issue, HaloTag, a 297 amino acid tag derived from a bacterial haloalkane dehalogenase was designed that offers an important advantage by formation of a highly specific covalent bond with its synthetic ligands [18]. This property allows immobilization and manipulation of bait proteins even under stringent buffer conditions. In order to offer a HaloTag bait protein-coding vector, the Halo protein coding sequence was inserted upstream of the TEV protease cleavage recognition motif and the LIC site (Fig. 3a). To check the functionality of the created vector, Halo-AtMPK9 protein was translated and purified by HaloTag affinity beads. Since the strong covalent bond formed by the dehalogenase enzyme was not cleavable even by boiling the sample in SDS loading buffer (data not shown), AtMPK9 was released from the beads by TEV protease treatment. Western blot analysis of total translation mixture and supernatant of TEV treated beads by AtMPK9 selective antibody demonstrated the success of the vector construction: a single protein of total translation mixture was decorated at the expected molecular weight, and according to the Western blot results, the covalently bead-bound Halo-AtMPK9 was recovered from the beads by TEV protease cleavage since a single protein with the molecular weight of AtMPK9 was detected in the supernatant by anti-MPK9C antibody (Fig. 3b).

**Fig. 4:**
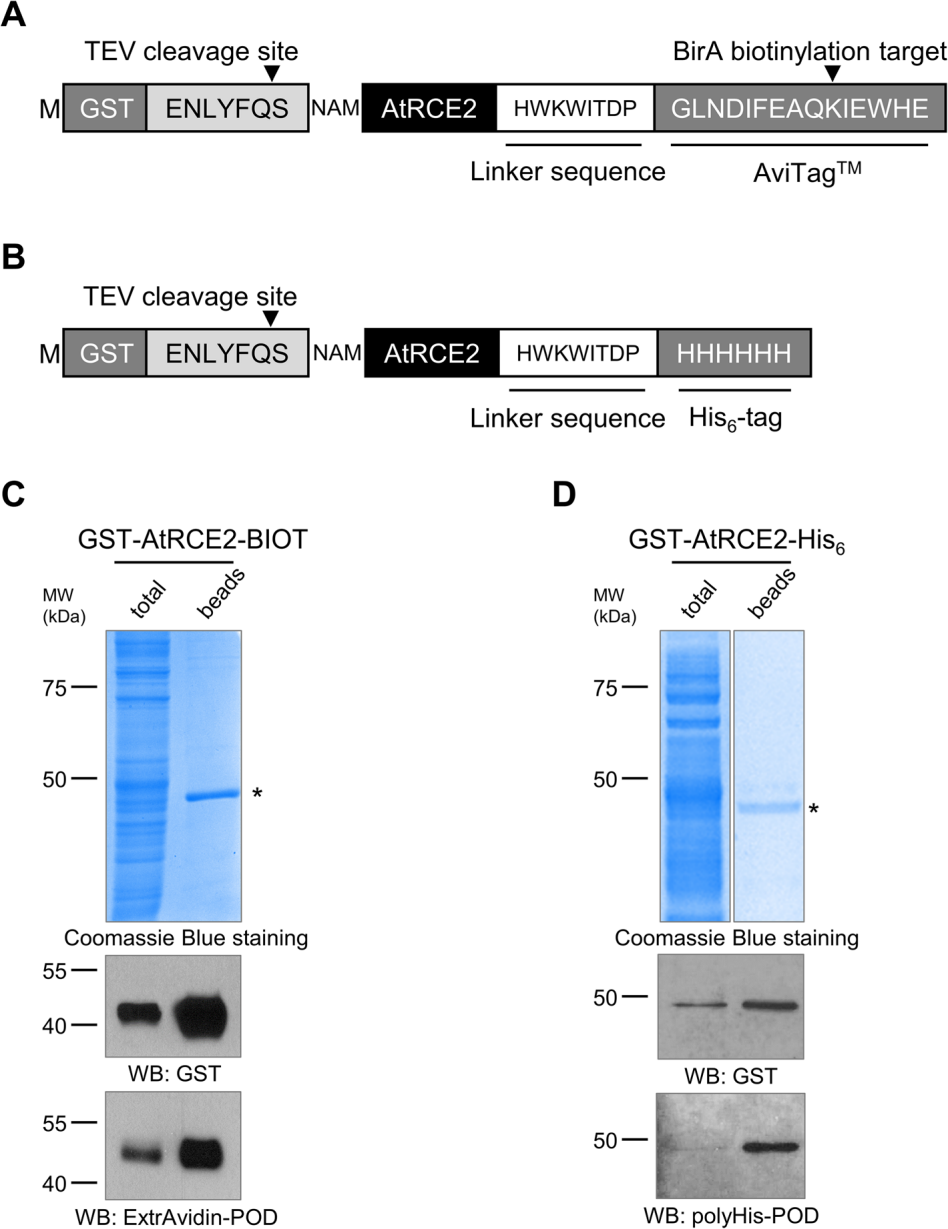
Western blot analysis of purified GST-AtRCE2-BIOT and GST-AtRCE2-His_6_ double-tagged proteins. **a** The amino acid sequence of the tagging region of GST-AtRCE2-BIOT. **b** The amino acid sequence of the tagging region of GST-AtRCE2-His_6_. **c**, **d** The double-tagged proteins (GST-AtRCE2-BIOT and GST-AtRCE2-His_6_) were purified by glutathione affinity beads. The samples were separated by SDS-PAGE, visualized by Coomassie Blue staining and Western blotting with anti-GST, and polyHis-POD antibodies or ExtrAvidin-POD. Affinity purified proteins are marked by asterisks. The molecular weight markers are shown in kDa

The most generally used method to produce purified and tagged recombinant proteins is the elution of proteins by solutions of ligand binding site competing compounds. However, the presence of added compounds in the eluate could often hinder the downstream processes; hence, they have to be removed by dialysis or desalting. The above-mentioned steps necessarily result in some loss of protein of interest making purification of protein at low concentration extremely challenging. Double-labelled recombinant protein coding vectors could be valuable tools in the above scenarios assuming that one of the tags can be cleaved by protease digestion. In consideration of this advantage of double-labelled proteins, we set out to further modify our GST-tag containing vector by the insertion of His_6_-tag or AviTag biotin labelling sequence [19] downstream of the LIC site (Fig. 4a and b). The created novel vectors were assessed by applying them to produce AtRCE2, an *Arabidopsis thaliana* ubiquitin-conjugating enzyme. The proteins were in vitro translated as described above and the AviTag biotinylation was accomplished co-translationally. To this end, the translation mixture was supplemented with biotin and BirA biotin ligase enzyme. Following 20 h of translation, the proteins were purified by using glutathione affinity beads. PAGE separation and Coomassie Blue staining of bead-bound fraction showed a single protein of estimated size implying successful GST-tagging and effective purification of AtRCE2 (Fig. 4c and d). To testify the presence of C-terminal His_6_ and biotin label extensions of synthesized AtRCE2, both total translation mixtures and glutathione bead-bound fractions were analysed by using peroxidase conjugated anti-polyHistidine antibody and ExtrAvidin. The peroxidase generated chemiluminescence signal provided a single band at the expected molecular weight with both tags indicating successful creation of double-tagging vectors (Fig. 4c and d).

## Conclusions

Here, we presented the improvement of the previously constructed pEU3-NII ligation-independent cloning plasmids and the creation of a wheat germ cell-free translation compatible vector family with a variety of affinity tags. Insertion of NotI - a rare-cutting restriction enzyme - recognition site to the 3′-UTR region evades extended restriction endonuclease site analysis of vector constructs thus allows general handling of multiple constructs simultaneously. The application of developed vector family provides various labelling opportunities for the protein of interest. The N-terminal His_6_- or FLAG-tag coding vectors could be respectively applied in experimental arrangements when sensitivity or selectivity has the highest priority. The HaloTag possessing vector could be a rational choice for synthesizing proteins for pull-down assays since the Halo provided covalent linkage of bait protein permits application of stringent washing conditions. The double-labelled GST-His_6_ and GST-biotin holding vectors have two advantageous features. First, the N-terminally localized GST- tag helps folding of the protein of interest; thus, it could help the production of challenging proteins. Second, due to TEV cleavability of GST, the affinity-tagged proteins could be purified without using buffers that could hinder the downstream application of the protein of interest. Summarily, the described vector family could present a useful toolkit for in vitro protein studies by providing a straightforward approach for producing proteins of various labels.

## Methods

### Cloning

The pEU3-NII vector backbone was previously modified by Bardóczy et al. [5] to encode for His_6_-tag (pEU3-NII-HLIC) and GST-tag (pEU3-NII-GLIC). The NotI restriction endonuclease was introduced into both vectors using QuikChange Site-Directed Mutagenesis Kit (Agilent) according to the provided manual with the 5′-ATTCTGAGAATAGTGTATGCGGCcgCCGAGTTGCTCTTGCCCGG-3′ and 5′-CCGGGCAAGAGCAACTCGGcgGCCGCATACACTATTCTCAGAAT-3′ primers (NotI recognition sequence underlined, mutated bases lowercase). The resulting pEU3-NII-HLICNot and pEU3-NII-GLICNot vectors were further manipulated to produce novel modified vectors suitable for wheat germ cell-free protein expression.

In general, the previously constructed pEU3-NII-HLICNot and pEU3-NII-GLICNot vectors were cut with the appropriate FastDigest restriction endonucleases (Thermo Scientific), treated with FastAP Thermosensitive Alkaline Phosphatase (Thermo Scientific) and used in ligation reactions. The colonies containing the new fragments were selected by colony PCR with pEU3-NII sequencing primers pEU3-NII-forward (5′-CACTATAGGGTACACGGAATTCGC-3′) and pEU-rev (5′-TATAGGAAGGCCGGATAAGACG-3′). Colony PCR positive clones were propagated, and the plasmid DNAs were isolated with Zippy Plasmid Miniprep kit (Zymo Research) for sequencing. See Supplementary Figure 1, Additional file 1 for the schematic representations of vector constructs.

#### pEU3-NII-HxHLICNot

The SpeI-linker-hexaHistidine-SpeI encoding fragment was generated by annealing oligonucleotides: 5′-CGGACTAGTATGCATCATCATCATCATCATTCTCGTGCTTGGCGTCACCCGCAGTTCGGTGGTACTAGTCCG-3′ and 5′-CGGACTAGTACCACCGAACTGCGGGTGACGCCAAGCACGAGAATGATGATGATGATGATGCATACTAGTCCG-3′. The created fragment was digested with SpeI and inserted into SpeI-digested pEU3-NII-HLICNot.

#### pEU3-NII-FLAGLICNot and pEU3-NII-HaloLICNot

The FLAGTEVLIC encoding cassette harbouring SpeI and SspI recognition sites was generated by annealing oligonucleotides 5′-GGACTAGTATGGATTATAAGGACGACGACGACAAAGAGAACCTGTACTTCCAATCCAATATTGG-3′ and 5′-CCAATATTGGATTGGAAGTACAGGTTCTCTTTGTCGTCGTCGTCCTTATAATCCATACTAGTCC-3′. The HaloTag sequence including the TEV protease recognition site was amplified with forward primer 5′-TAACTAGTATGGCAGAAATCGGTACTG-3′ and reverse primer 5′-ATTGGATTGGAAGTACAGATCCTCAGTGG-3′ from the pHTN HaloTag® CMV-neo Vector (Promega) so that SpeI restriction endonuclease recognition sequence and SspI blunt end sequence were appended to both ends of the PCR product. Both FLAG and Halo coding DNA sequences were digested with the appropriate enzymes and inserted into SpeI and SspI digested pEU3-NII-GLICNot vector.

#### pEU3-NII-GLICNot-C-BIOT and pEU3-NII-GLICNot-C-his

The C-terminal AviTag and His_6_-tag encoding cassettes with BamHI and SmaI overhangs were generated by annealing the following oligonucleotides:

- AviTag
  5′-AAGGATCCCGGCCTCAACGACATCTTCGAGGCCCAGAAGATCGAGTGGCACGAGTGACCCGGGT-3′ and 5′-ACCCGGGTCACTCGTGCCACTCGATCTTCTGGGCCTCGAAGATGTCGTTGAGGCCGGGATCCTT-3′;
- His_6_-tag
  5′-AAGGATCCCCATCATCATCATCATCATTGACCCGGGT-3′ and 5′-ACCCGGGTCAATGATGATGATGATGATGGGGATCCTT-3′.

The obtained dsDNA fragments were treated with the appropriate enzymes and inserted into BamHI and SmaI digested pEU3-NII-GLICNot vector.

### Ligation-independent cloning

Protein coding sequences were inserted into the appropriate vectors by ligation-independent cloning (LIC) according to the previously described method [20]. AtMPK9 (AT3G18040) was inserted into pEU3-NII-HLICNot, pEU3-NII-HxHLICNot, pEU3-NII-FLAGLICNot, and pEU3-NII-HALOLICNot. AtRCE2 (AT2G18600) was inserted into pEU3-NII-GLICNot-C-BIOT and pEU3-NII-GLICNot-C-His.

Briefly, the inserts were amplified by PCR with their specific forward and reverse primers (See Supplementary Table 1, Additional file 2) and purified by polyethylene glycol and ethanol precipitation. The vectors were linearized with SspI and purified from agarose gel following electrophoresis. The SspI cut vectors were incubated with dGTP (Bioline) and the inserts were incubated with dCTP (Bioline) in the presence of T4 DNA polymerase (Roche). The T4 treated vectors and inserts were mixed at a 1:3 molar ratio in the presence of EDTA and the resulting ligation mixtures were transformed into XL10-Gold competent cells. Colony PCR positive clones were propagated, and the plasmid DNAs were isolated with Zippy Plasmid Miniprep kit (Zymo Research).

### In vitro transcription and translation

The vector constructs were used for in vitro transcription and translation as described previously [20]. Briefly, the vectors were linearized by NotI restriction endonuclease enzyme, purified by polyethylene glycol and ethanol precipitation; 1 μg of linearized plasmid was used as a template of in vitro transcription reaction performed by TranscriptAid T7 High Yield Transcription Kit (Thermo Scientific) according to the manufacturer’s protocol. The mRNA was purified by ammonium acetate and ethanol precipitation and resuspended in 20 μl of 1X SUB-AMIX solution (CellFree Sciences). 5 μl WEPRO wheat germ extract (CellFree Sciences, W7240G except for the His-tagged proteins that were translated with W7240H or W7240 extract, as indicated), 2.5 μl SUB-AMIX, 2.5 μl mRNA, 0.4 μl 1 mg/ml creatine kinase (Roche) were gently mixed and carefully underlaid to 103 μl SUB-AMIX solution in a 96-well plate. AviTag containing proteins were biotinylated during the translation reactions by adding 25 ng Trx-BirA biotin ligase enzyme [21] to the bottom layer, and 0.5 μM (final concentration) of D-biotin (Supelco) to both layers [22]. The translation reactions were carried out for 20 h at 20 °C, that yielded approximately 5–10 μg protein of interest in 113.4 μl total reaction mixture. Expected molecular weights of expressed proteins: His_6_-AtMPK9 60.4 kDa, His_12_-AtMPK9 62.8 kDa, FLAG-AtMPK9 60.6 kDa, Halo-AtMPK9 93.5 kDa, GST-AtRCE2-BIOT 50.8 kDa, GST-AtRCE2-His_6_ 49.8 kDa.

### Purification of expressed proteins

#### Purification of His_6_-AtMPK9 and His_12_-AtMPK9

One hundred microliter translation mixture was diluted two times with 2X Lysis Buffer (100 mM NaH_2_PO_4_, 600 mM NaCl, 20 mM imidazole, pH 8), mixed with 13 μl equilibrated PureCube Co-NTA Agarose (Cube Biotech) and incubated on a rotator for 1 h at room temperature. After washing the beads three times with Wash Buffer (50 mM NaH_2_PO_4_, 300 mM NaCl, 20 mM imidazole, pH 8), the beads were resuspended in 50 μl PBS.

#### Purification of HALO-AtMPK9

Twenty microliter translation mixture was diluted two times with HALO Equilibration buffer (PBS with 0.005% NP-40), mixed with 0.5 μl equilibrated Magne HaloTag Beads (Promega) and incubated on a rotator for 1 h at room temperature. After washing the beads three times with HALO Equilibration buffer, the beads were resuspended in 10 μl 1X TEV buffer (Sigma) containing 0.1 μl TEV protease (Sigma, T4455) and 1 mM DTT, and the cleavage reaction was conducted for 1 h at 30 °C. The supernatant containing the untagged AtMPK9 with two extra amino acids at its N-terminal (expected molecular weight 58.7 kDa) was mixed with SDS-DTT sample buffer and boiled for 5 min at 95 °C.

#### Purification of GST-AtRCE2-BIOT and GST-AtRCE2-His_6_

One hundred microliter translation mixture was diluted six times with Binding/Wash Buffer (125 mM Tris-HCl pH 8, 150 mM NaCl, 0.5% Triton-X), mixed with 12.5 μl equilibrated Pierce Glutathione Magnetic Beads (Thermo Scientific) and incubated on a rotator for 1 h at room temperature. The beads were washed three times with Binding/Wash buffer and resuspended in 50 μl PBS.

### SDS-PAGE and Western blot

The samples were mixed with SDS-DTT sample buffer and boiled for 5 min at 95 °C. If not indicated otherwise, 5 μl of total translation mixture and 10 μl of bead-bound proteins were prepared for Coomassie Blue staining, and 1–3 μl of total translation mixture and 2–5 μl of bead-bound proteins were prepared for immunoblotting. The protein samples were separated on 8%/10%/15% SDS-PAGE with Laemmli buffer or 4–12% Bis-Tris gradient gels (Expedeon) with MES buffer (Expedeon) and the proteins were either stained directly in the gel by Coomassie Blue or transferred to PVDF membrane by semi-dry blotting. The protein samples, which were separated on 10% TGX Stain-Free Precast gels with Laemmli buffer, were activated and imaged with Gel Doc XR+ Gel Documentation System (Bio-Rad) and subsequently transferred to PVDF membrane by semi-dry blotting. The membranes were blocked with 5% non-fat milk powder in PBS/TBS containing 0.05% Tween-20. The proteins were detected either directly by anti-polyHistidine-POD (Sigma) diluted 1:2000 or by rabbit anti-GST (Upstate) diluted 1:2000, rabbit anti-MPK9C (raised in-house) diluted 1:2000, mouse anti-FLAG® M2 (Sigma) diluted 1:2000 and secondary antibodies goat anti-rabbit-HRP (Cell Signaling) and horse anti-mouse-HRP diluted 1:5000. To detect biotinylated proteins, the membranes were blocked with 5% BSA in PBS containing 0.05% Tween-20 and challenged with 1:5000 diluted ExtrAvidin-POD (Sigma). The peroxidase activity was detected by ECL (Thermo Scientific) in all cases.

### Accession numbers

The created vectors are available via Addgene by using the following ID numbers:

pEU3-NII-HLICNot (ID 140181), pEU3-NII-GLICNot (ID 140182), pEU3-NII-HxHLICNot (ID 140183), pEU3-NII-FLAGLICNot (ID 140184), pEU3-NII-HaloLICNot (140185), pEU3-NII-GLICNot-C-BIOT (ID 140186), pEU3-NII-GLICNot-C-His (140187).

## Supplementary information

**Additional file 1 : Supplementary Figure 1**. Schematic illustrations of modified pEU3-NII plasmids. Utilized restriction endonucleases (the unique cutters are shown in bold), plasmid names and sizes are indicated on the maps. Ampicillin resistance gene (AmpR), origin of replication (ori), T7 promoter, glutathione-S-transferase (GST) gene, tobacco etch virus protease recognition site (TEV site with amino acid sequence E(N/D)LYFQS), LIC (ligation-independent cloning) site. Plasmid maps produced by using SnapGene software (from GSL Biotech; available at snapgene.com).

**Additional file 2 Supplementary Table 1.** Sequences of primers used for ligation-independent cloning. Underlined letters represent the start and stop codons, bold letters the gene-specific sequences.

## Data Availability

All data generated or analysed during this study are included in this published article and Additional files 1 and 2.

## Abbreviations

AtMPK9: *Arabidopsis thaliana* mitogen-activated protein kinase 9
AtRCE2: *Arabidopsis thaliana* related to ubiquitin-conjugating enzyme 2
GST: Glutathione S-transferase
His_12_: Double-His_6_
His_6_: Hexahistidine
IMAC: Immobilized metal affinity chromatography
LIC: Ligation-independent cloning
Ni-NTA: Nickel-nitrilotriacetic acid
PVDF: Polyvinylidene fluoride
TEV: Tobacco etch virus
