# Supplementary figures and images for: A novel family of expression vectors with multiple affinity tags for wheat germ cell-free protein expression
Source: BMC Biotechnol. 2020 Mar 14;20:17. doi: 10.1186/s12896-020-00610-5 (PMC7071761; doi:10.1186/s12896-020-00610-5)

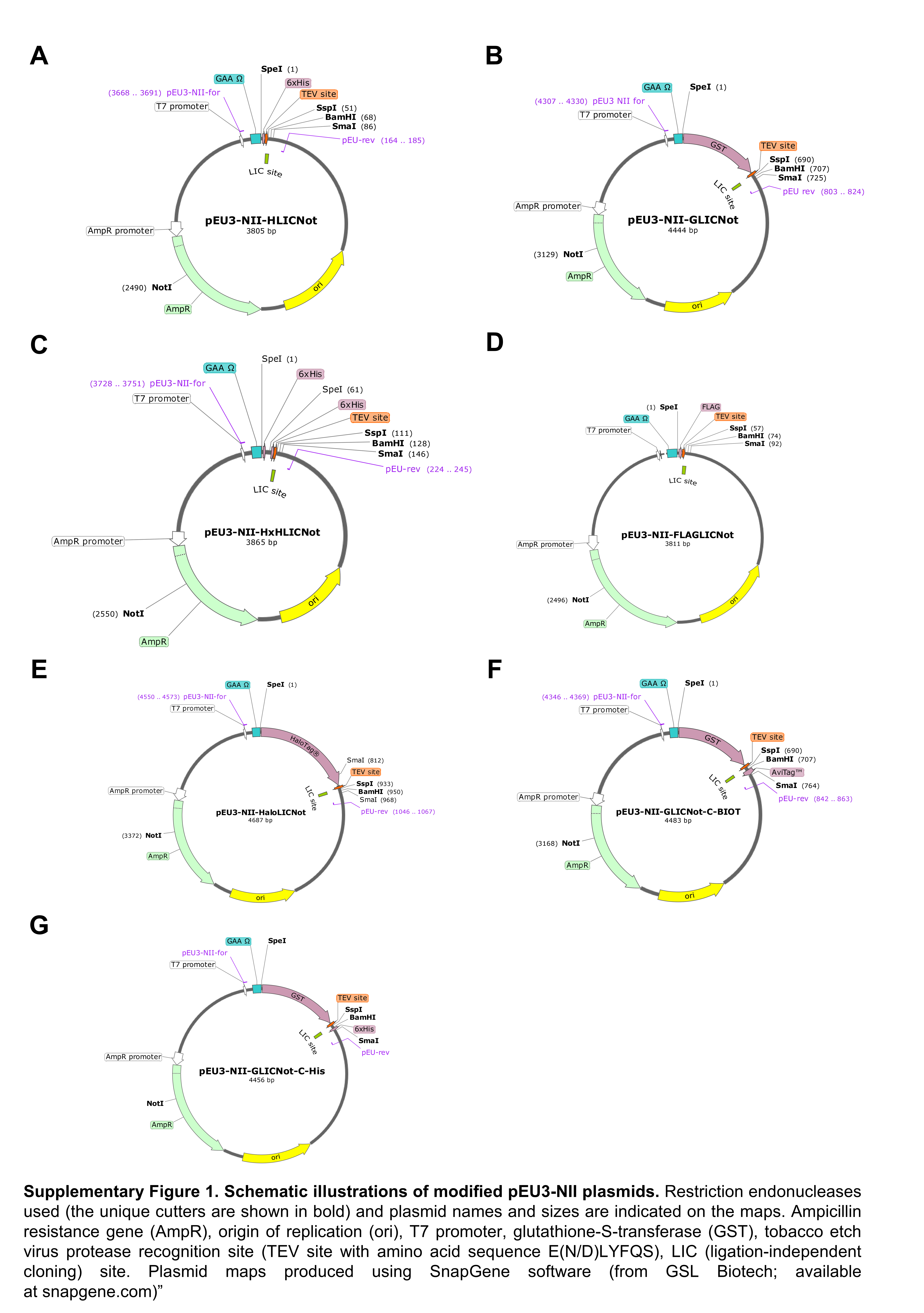

Supplement: Supplementary file 1 — Additional file 1 : Supplementary Figure 1. Schematic illustrations of modified pEU3-NII plasmids. Utilized restriction endonucleases (the unique cutters are shown in bold), plasmid names and sizes are indicated on the maps. Ampicillin resistance gene (AmpR), origin of replication (ori), T7 promoter, glutathione-S-transferase (GST) gene, tobacco etch virus protease recognition site (TEV site with amino acid sequence E(N/D)LYFQS), LIC (ligation-independent cloning) site. Plasmid maps produced by using SnapGene software (from GSL Biotech; available at snapgene.com). [file 12896_2020_610_MOESM1_ESM.tif]
