# Supplementary material for: A novel family of expression vectors with multiple affinity tags for wheat germ cell-free protein expression
Source: BMC Biotechnol. 2020 Mar 14;20:17. doi: 10.1186/s12896-020-00610-5 (PMC7071761; doi:10.1186/s12896-020-00610-5)
Supplement: Supplementary file 2 — Additional file 2 Supplementary Table 1. Sequences of primers used for ligation-independent cloning. Underlined letters represent the start and stop codons, bold letters the gene-specific sequences. [file 12896_2020_610_MOESM2_ESM.pdf]

Supplementary Table 1. Sequences of primers used for ligation-independent cloning. Underlined letters represent the start and stop codons, bold letters the gene-specific sequences.

Primer name	Sequence (5'→3')
AtMPK9-LICfor	TACTTCCAATCCAATGCA<u>ATG</u>**GATCCTCATAAAAAGGTTG**
AtMPK9-LICrev	TTATCCACTTCCAATG<u>TCA</u>**AGTGTGGAGAGCCGC**
AtRCE2-LICfor	TACTTCCAATCCAATGCA<u>ATG</u>**ATTGGGTTATTCAAAGTGAAA**
AtRCE2-LICrev	TTATCCACTTCCAATG<u>TCA</u>**CATGCAGCGAGGAAATGAGGT**
